# Comparative analysis of chromosomes in the Palaearctic bush-crickets of tribe Pholidopterini (Orthoptera, Tettigoniinae)

**DOI:** 10.3897/CompCytogen.v11i2.12070

**Published:** 2017-05-05

**Authors:** Elżbieta Warchałowska-Śliwa, Beata Grzywacz, Klaus-Gerhard Heller, Dragan P. Chobanov

**Affiliations:** 1 Institute of Systematics and Evolution of Animals, Polish Academy of Sciences, Sławkowska 17, 31-016 Krakow, Poland; 2 Grillenstieg 18, 39120 Magdeburg, Germany; 3 Institute of Biodiversity and Ecosystem Research, Bulgarian Academy of Sciences, 1 Tsar Osvoboditel Boul., 1000 Sofia, Bulgaria

**Keywords:** Orthoptera, Pholidopterini, karyotype, FISH, 18S rDNA, telomeric DNA, NOR, C-banding, fluorochrome staining

## Abstract

The present study focused on the evolution of the karyotype in four genera of the tribe Pholidopterini: *Eupholidoptera* Mařan, 1953, *Parapholidoptera* Mařan, 1953, *Pholidoptera* Wesmaël, 1838, *Uvarovistia* Mařan, 1953. Chromosomes were analyzed using fluorescence *in situ* hybridization (FISH) with 18S rDNA and (TTAGG)*_n_* telomeric probes, and classical techniques, such as C-banding, silver impregnation and fluorochrome DAPI/CMA_3_ staining. Most species retained the ancestral diploid chromosome number 2n = 31 (male) or 32 (female), while some of the taxa, especially a group of species within genus *Pholidoptera*, evolved a reduced chromosome number 2n = 29. All species show the same sex determination system X0/XX. In some taxa, a pericentric inversion has changed the morphology of the ancestral acrocentric X chromosome to the biarmed X. The rDNA loci coincided with active NORs and C-band/CG-rich segments. A comparison of the location of the single rDNA/NOR in the genus *Pholidoptera* suggests that reduced chromosome number results from Robertsonian translocation between two pairs of autosomes, one carrying the rDNA/NOR. The results constitute a step towards better understanding of the chromosomal reorganization and evolution within the tribe Phaneropterini and the whole subfamily Tettigoniinae.

## Introduction

Tettigoniinae Krauss, 1902 is amongst the largest groups of Tettigoniidae (also known as the bush-crickets or the katydids) with over 500 species occurring over five continents with the exception of the equatorial and subequatorial climatic zones (Rentz and Colless 1990, Cigliano et al. 2017). The Tettigoniinae has been considered by different authors with different systematic rank or taxonomic composition, being treated also as a family (e.g. Kevan 1982, Heller 1988). Recently, it has mostly been regarded as a subfamily including 12 tribes (Cigliano et al. 2017). However, the present tribal composition and ranking have been a matter of diverse interpretation (e.g. Gorochov 1995, Massa and Fontana 2011).

Ramme (1951) first established the group “Pholidopterini” based mostly on a comparison of the male copulatory structures including the genera *Apholidoptera* Mařan, 1953, *Parapholidoptera* Mařan, 1953, *Pholidoptera* Wesmaël, 1838, *Uvarovistia* Mařan, 1953 (the latter validated according to the ICZN by Mařan 1953). Later, the genus *Exopholidoptera* has been added by Ünal (1998).

The diploid chromosome number (2n), chromosome morphology (FN), and type of sex determination systems of Tettigoniinae have been described for more than 100 species out of 35 genera. Most of the Old World species have 31 acrocentric chromosomes, but the few New World genera diverge from this standard karyotype with one or more pairs of metacentric autosomes and often metacentric X chromosome. Hence, the karyotypes in this group range from 23 to 33 in male (reviewed in Warchałowska-Śliwa 1998).

Molecular cytogenetic methods through mapping molecular markers such as repetitive or unique sequences on chromosomes have been successfully applied for interspecific comparative karyotype structure and evolution in insects, especially coleopterans (e.g. Cabral-de Mello et al. 2011a), lepidopterans (e.g. Nguyen et al. 2010, Vershinina et al. 2015) hemipterans (Kuznetsova et al, 2015, Maryańska-Nadachowska et al. 2016), and orthopterans (grasshoppers e.g. Cabrero et al. 2009, Cabral-de-Mello 2011b, Jetybayev et al. 2012, Neto et al. 2013). Recently, a series of works using advanced, such as fluorescence *in situ* hybridization (FISH), and conventional chromosome banding techniques, showed that the number and location of rDNA (NOR) and heterochromatin, respectively, can be useful markers for the studying the karyotypes evolution in tettigoniids, and for the identification of genus/species-specific patterns, namely in the subfamilies Phaneropterinae (e.g. Warchałowska-Śliwa et al. 2011, 2013a, Grzywacz et. al. 2011, 2014a,b), Saginae (Warchałowska-Śliwa et al. 2009), Hetrodinae (Warchałowska-Śliwa et al. 2015) and Bradyporinae (Warchałowska-Śliwa et al. 2013b). However, data on NORs in Tettiginiinae genomes are scarce (Warchałowska-Śliwa et al. 2005) and no mapping location of rDNA clusters and telomeric DNA have been done so far.

Until now, the karyotypes of 16 species/subspecies of Pholidopterini, occasionally examined using C-banding and NOR staining have been studied (Warchałowska-Śliwa et al. 2005). For the first time, we performed cytogenetic analysis of the chromosome number of 27 Pholidopterini taxa belonging to four genera (*Eupholidoptera* Mařan, 1953, *Parapholidoptera* Mařan, 1953, *Pholidoptera* Wesmaël, 1838, *Uvarovistia* Mařan, 1953). We examined the number and distribution of rDNA clusters in 28 taxa by FISH with 18S rDNA and telomeric (TTAGG)*_n_* probes as well as by conventional methods with the aim to estimate the role of analyzed markers of understanding genome organization in this tribe.

## Material and methods

A total of 74 specimens of the genera *Eupholidoptera*, *Parapholidoptera*, *Pholidoptera* and *Uvarovistia* (Pholidopterini), belonging to 38 species/subspecies were studied cytologically. Table 1 summarizes the geographic origin and cytogenetic-molecular data of the analyzed material (including data reported earlier). Testes and ovarioles were incubated in a hypotonic solution (0.9% sodium citrate), then fixed in ethanol - acetic acid (3:1) and squashed in a drop of 45% acetic acid. The cover slip was removed using the dry ice procedure. Slides were dehydrated and air dried. Chromosome preparation for the C-banding examination was carried out according to Sumner (1972). Constitutive heterochromatin was analyzed qualitatively by CMA_3_ (chromomycin A_3_) and DAPI (4,6-diamidino-2-phenylindole) staining according to Schweizer (1976). Silver nitrate staining for active nucleolus organizing regions (NORs) was achieved using the protocol of Warchałowska-Śliwa and Maryańska-Nadachowska (1992).

The best chromosome preparations were used for fluorescence *in situ* hybridization (FISH) with 18S ribosomal DNA (rDNA) and telomeric DNA (TTAGG)*_n_*. FISH was carried out as described earlier in Warchałowska-Śliwa et al. (2009). For detection of rDNA clusters in chromosomes, a probe containing a fragment of orthopteran 18S rDNA labeled with biotin-16-dUTP was used. In order to study the organization of telomeric repeats (TTAGG)*_n_* in chromosomes, a DNA probe was generated by non-template PCR with Tel1 [5’ GGT TAG GTT AGG TTA GGT TAG G 3’] and Tel2 [5’ TAA CCT AAC CTA ACC TAA 3’] primers. Visualization of hybridized DNA labeled with biotin and digoxigenin was performed with sheep avidin-FITC conjugates and anti-digoxigenin-rhodamine, respectively. The chromosomes were counterstained with DAPI solution under a cover glass. Chromosomes were studied with a Nikon Eclipse 400 microscope with a CCD DS-U1 camera and NIS-Elements BR2. For each individual (analyzed for the first time) at least five oogonial/spermatogonial mitotic metaphase and 15 meiotic divisions (in male) were examined.

**Table 1.** Pholidopterini taxa: collection sites, comparison of the number and morphology of chromosomes, distribution of rDNA cluster and NOR.

Species	Collection sites	Geographical coordinates	No.	2n, FN	References	rDNA-NOR
***Eupholidoptera*** *anatolica* (Ramme, 1930)	TR: Antalya, Termessos	36°58'N, 30°30'E	2m	31,31	Warchałowska-Śliwa et al. 2005	no
*Eupholidoptera annulipes* (Brunner von Wattenwyl, 1882)	TR: Mersin, below Güzeloluk	36°44'N, 34°9'E	2m	31,31	Warchałowska-Śliwa et al. 2005	no
*Eupholidoptera astyla* (Ramme, 1927)	GR: 1) Crete, Rethimni, Mt. Ida, spring of Skaronero, above Kamares	1) 35°10'N, 24°48'E	1m	31,31	this study	3/4i-3/4i
	2) Crete, Rethimini, Skaleta	2) 35°24'N, 24°37'E	1m			
*Eupholidoptera epirotica* (Ramme, 1927)	GR: Aitolia-Akarnania (Central Greece), Mt. Karnania above Thirion	38°48'N, 20°58'E	6m, 1f	31,31	Warchałowska-Śliwa et al. 2005	no-3/4i
*Eupholidoptera chabrieri garganica* (La Greca, 1959)	GR: Kerkyra, near Agios Spiridon	39°48'N, 19°50'E	3m	31,31	Warchałowska-Śliwa et al. 2005	no
*Eupholidoptera giuliae* Massa, 1999	GR: Crete, Chania, Chora Sfakion	35°12'N, 24°8'E	3m	31,31	this study	no
*Eupholidoptera icariensis* Willemse, 1980	GR: Samos, Ikaria	37°36'N, 26°9'E	1m	31,31	Warchałowska-Śliwa et al. 2005	no
*Eupholidoptera karabagi* Salman, 1983	GR: Antalya, Termessos	36°58'N, 30°30'E	3m	31,31	Warchałowska-Śliwa et al. 2005	no-3/4i
*Eupholidoptera krueperi* (Ramme, 1930)	TR: Antalya, Kozdere Köprü	36°36'N, 30°31'E	1m	31,31	this study	3/4i-3/4i
*Eupholidoptera latens* Willemse & Kruseman, 1976)	GR: Crete, Chania, Rodopos	35°33'N, 23°45'E	2m	31,31	this study	no
*Eupholidoptera megastyla* (Ramme, 1939)	GR: 1) Myrsini, Pd Peloponez	39°25'N, 21°10'E	4 m	31,31	this study	3/4i-3/4i
	2) Arta, Tsoumerka Mt.		2m		Warchałowska-Śliwa et al. 2005	no
*Eupholidoptera mersinensis* Salman, 1983	TR: 1) Mersin, Fýndýkpýnarý	1) 36°50'N, 34°20'E	1m	31,31	Warchałowska-Śliwa et al. 2005	no 3/4i
	2) Mersin, below Güzeloluk (near Köserlý)	2) 36°45'N, 34°7'E	1m			
*Eupholidoptera prasina* (Brunner von Wattenwyl, 1882)	TR: Mersin, Kasyayla (above Anamur)	36°15'N, 32°54'E	5m	31,31	Warchałowska-Śliwa et al. 2005	no
*Eupholidoptera schmidti* (Fieber, 1861)	MK: 1) Gorna Belica	1) 41°13'N, 20°33'E	2m	31,31	this study	3/4i-3/4i
	2) Korab Mt.	2) 41°41'N, 20°39'E	1m			
*Eupholidoptera smyrnensis* (Brunner von Wattenwyl, 1882)	1) TR: N Sindrigi	1) 39°18'N, 28°12'E	1m	31,31	this study	3/4i-3/4i
	2) BG: Melnik	2) 41°31'N, 23°23'E	2m			
*Eupholidoptera tauricola* (Ramme, 1930)	TR: Mersin, below Güzeloluk	36°44'N, 34°9'E	2m	31,31	Warchałowska-Śliwa et al. 2005	no
*Eupholidoptera tahtalica* (Uvarov, 1949)	TR: Aspendos close Antalya	36°56'N, 31°10'E	1m	31,31	this study	3/4i-3/4i
***Parapholidoptera*** *castaneoviridis* (Brunner von Wattenwyl, 1882)	BG: 1) Stara Planina Mts, Karandila site	1) 42°43'N, 26°22'E	2m	31,32	this study	3/4i-3/4i
	2) Strandzha Mts, Kovach place	2) 42°05'N, 27°25'E	1m			
Parapholidoptera cf. belen Ünal, 2006	TR: Yiglica	40°58'N, 31°34'E	1m	31,31	this study	3/4i-3/4i
*Parapholidoptera distincta* (Uvarov, 1921)	GE: E Nailevi	41°38'N, 42°35'E	2m	31,31 +B	this study	3/4i-3/4i
*Parapholidoptera noxia* (Ramme, 1930)	GE: 1) Gombori range	1) 41°52'N, 45°18'E	1m	31,31	this study	3/4i-3/4i
	2) SW of Gora vill.	2) 41°14'N, 44°17'E	2m			
*Parapholidoptera grandis* (Karabag, 1952)	TR: E Ibradi	37°03'N, 31°44'E	4m	31,32	this study	3/4i-3/4i*
*Parapholidoptera signata* (Brunner von Wattenwyl, 1861)	TR: Mersin, below Güzeloluk	36°44'N, 34°9'E	1m	31,31	Warchałowska-Śliwa et al. 2005	no
Parapholidoptera cf. signata	TR: above Zara	39°37'N, 37°56'E	1m	31,31	this study	no
Parapholidoptera aff. syriaca	TR: Demre	36°27'N, 30°26'E	3m	29,31	this study	3/4i-3/4i*
***Pholidoptera*** *dalmatica maritima/ebneri*	MO: Cetinje, Skader lake	42°23'N, 55°45'E	2m	31,31	this study	3/4i-3/4i
*Pholidoptera fallax* (Fischer, 1853)	1) BG: Veliko Tarnovo 2) MO: Cetinje, Skader lake	1) 43°05'N, 25°39'E 2) 42°23'N, 55°45'E	2m 2m	31,31	this study	3/4i-3/4i
*Pholidoptera frivaldszkyi* (Herman, 1871)	1) BG: Batak	1) 43°05'N, 25°39'E	3m		this study	3/4i- 3/4i
	2) BG: Iskar	2) 41°57'N, 24°12'E	1m	31,31	Warchałowska-Śliwa et al. 2005	no
	3) GR: Drama, ca. 5 km north of Elatia	3) 41°30'N, 24°18'E	1m			
*Pholidoptera griseoaptera* Mařan, 1953	1) CR: Chetyr Dag	1) 44°44'N, 34°19'E	1m	31,31	this study	3/4i-3/4i
	2) PL: Ojców National Park, Sąspowska valley	2) 50°15'N, 19°50'E	6m		Warchałowska-Śliwa et al. 2005	no-3/4i
*Pholidoptera littoralis* (Fieber, 1853)	BG: Belogradchik	43°38'N, 22°41'E	1m, 1f	31,31	this study	3/4i 3/4i
*Pholidoptera pustulipes* (Motschulsky, 1846)	CR: 1) Chetyr Dag	1) 44°44'N, 34°19'E	1m	31,31	this study	3/4i-3/4i*
	2) Karadag Reserve	2) 44°55'N, 35°12'E	1m, 1f			
*Pholidoptera aptera aptera* (Fabricius, 1793)	PL: Pieniny Mts, polana Walusiówka	49°25'N, 20°28'E	4m	29,31	Warchałowska-Śliwa et al. 2005	no-1p
*Pholidoptera aptera bulgarica* Mařan, 1953	BG: E Rhodopi Mts, Shturets vill	41°37'N, 25°32'E	1m	29,31	this study	1p – 1p
*Pholidoptera* cf. *apterabulgarica*	MK: Strumica, Cham Chiflik	41°25'N, 22°38'E	1f	30,32	this study	1p-1p
*Pholidoptera aptera karnyi* Ebner, 1908	BG: 1) Stara Planina Mts, Uzana place	1) 42°46'N, 25°14'E	2m 1m	29,31	this study	1p-1p
	2) Lyulin Mt.	2) 42°39'N, 23°12'E			Warchałowska-Śliwa 1988	no
*Pholidoptera brevipes* Ramme, 1939	BG: 1) Sakar Mt., Matochina vill;	1) 41°51'N, 26°33'E	1m	29,32	this study	3/4i-3/4i
	2) Gorska Polyana vill.	2) 42°06'N, 26°58'E	2m			
Pholidoptera aff. brevipes	TR: Coroglu	40°53'N, 32°57'E	1m	29,32	this study	3/4i-3/4i
*Pholidoptera macedonica* Ramme, 1928	1) AL: Galichica Mt., Pilcina	1) 40°54'N, 20°52'E	2m		this study	1p-1p
	2) MK: Nidzhe Mt.	2) 41°00'N, 21°41'E	2f	29,31		
	3) MK: Gorna Belica	3) 41°13'N, 20°33'E	1m			
	4) GR: Drama, near Elatia	4) 41°30'N, 24°18'E	2m		Warchałowska-Śliwa et al. 2005	no
*Pholidoptera rhodopensis* Maran, 1953	BG: 1) near Goce Delcev	1) 41°47'N, 23°33'E	2m	29,31	this study	1p-1p
	2) Pirin Mt. above Bansko	2) 41°46'N, 23°27'E	1m			
*Pholidoptera stankoi* Karaman, 1960	MK: Ribnicka Riv	41°42'N, 20°39'E	3m	29,31	this study	1p-1p
***Uvarovistia*** *satunini* (Uvarov, 1934)	TR: 1) Tunceli-Mazgirt	1) 39°04'N, 39°34'E	2m	31,31	this study	4/5i 4/5i
	2) Yanıkcay	2) 38°15'N, 42°54'E	1m			

Note: ALAL = Albania, BG = Bulgaria, CR = Crimea, GE = Georgia, GR = Greece, MK = Macedonia, MO = Montenegro, TR = Turkey, PL = Poland. No = numbers of individuals studied, m = male and f = female; FN = fundamental number of chromosome arms in male (except female of *Pholidoptera* cf. *apterabulgarica*); B = B chromosome; a slash between two numbers indicates imprecise identification of the pair of bivalents; *high or low signal of 18S rDNA probes between homologous chromosomes.

## Results

**Figure 1.**
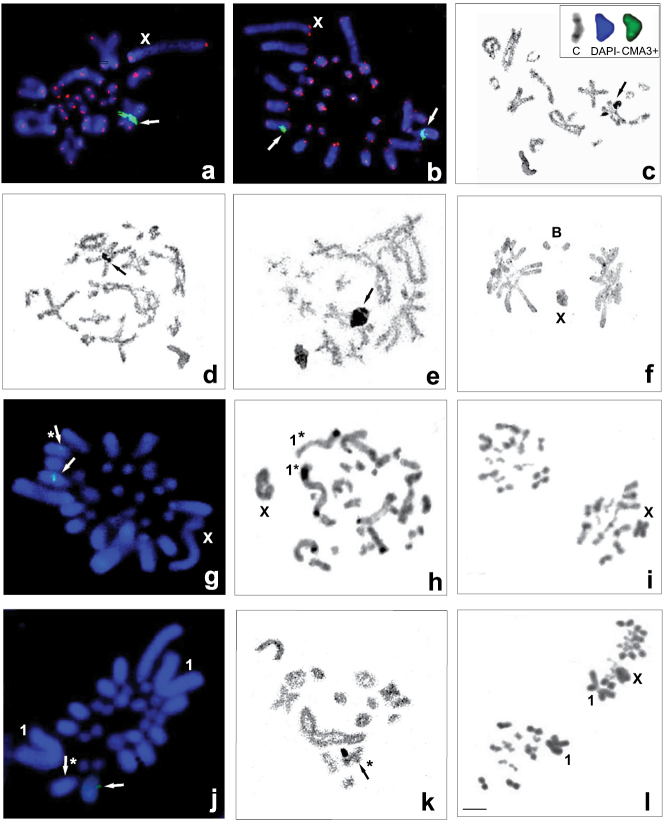
Examples of chromosome banding in different species of the tribes *Eupholidoptera* (**a–c**) and *Parapholidoptera* with 2n = 31 (**d–i**) and 2n = 29 (**j–l**) studied using different techniques. 18S rDNA FISH revealed a single interstitial cluster (per haploid genome) located in the 3/4 or 4/5 bivalent (**a, b, g, j**) and co-localized with the active NOR visualized by AgNO_3_ staining (**c–e, k**). **a** *E. astyla*, diakinesis and **b** *E. megastyla*, spermatogonial metaphase with 18S rDNA loci (green, arrows) located close to the paracentromeric region of bivalent 3/4 and telomeric DNA probes (red) **c** *E. schmidti*, diakinesis with NOR (arrow) and selected C+, DAPI- and CMA_3_+ bands located interstitially on 3/4 bivalent (in the right corner) **d** P. cf. belen and **e** *P. distincta*, diplotene, arrows indicate NOR located in the middle of bivalent 3/4 **f** *P. distincta*, anaphase I with B chromosomes **g, h, i** *P. grandis* **g** spermatogonial metaphase with the rDNA cluster with different size between homologous chromosomes (arrows) **h** heterochromatin heteromorphism in respect to the pattern of C-bands in the first autosome pair (1, asterisks) **i** two metaphase II with bi-armed X chromosome **j, k, l** P. aff. syriaca **j** spermatogonial metaphase, rDNA-FISH signal present/absent in homologous chromosomes (arrows and an asterisk) correspond to **k** NOR **l** two metaphase II with bi-armed first pair of autosomes (1) and acrocentric X. Bar = 10 µm.

**Figure 2.**
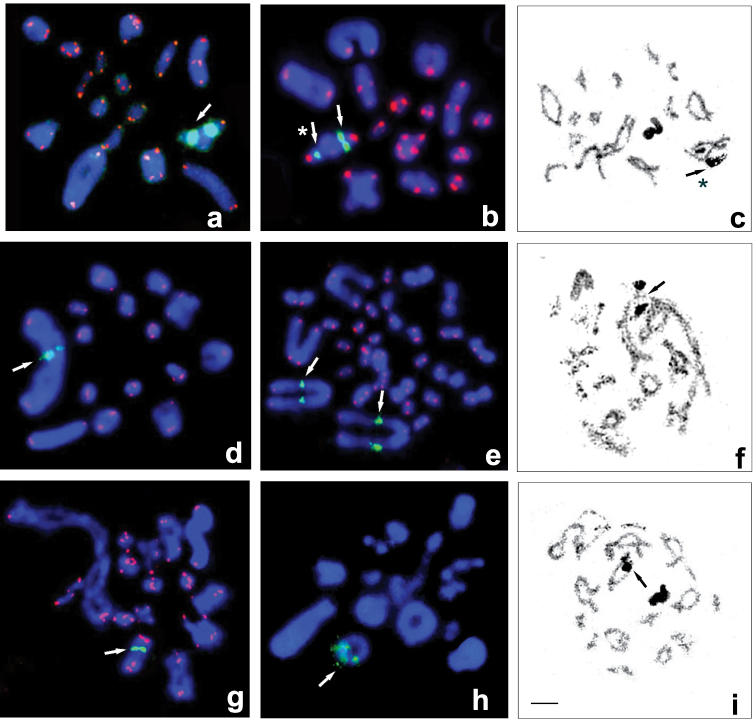
FISH using 18S rDNA (green) and telomeric TTAGG (red) probes on male (**a, b, d, g, h**) and female (**e**) karyotypes and silver staining (**c, f, i**). Diplotene of *Pholidoptera* (**a–g**) and *Uvarovistia* (**h, i**) species. White arrows point to rDNA clusters on the medium acrocentric pair or on the bi-armed first pair of autosomes. Black arrows indicate the active NOR co-localized with rDNA. **a** *P. fallax* and **b, c** *P. pustulipes* (2n = 31). Asterisks point to differences in size/strength of rDNA/NOR between homologous chromosomes **d–f** *P. macedonica* (2n = 29). Arrows indicate high-intensity rDNA signal and NOR located in the paracentromeric region of the bi-armed first pair of autosomes **g** *P. brevipes* (2n = 29). Arrows point 18S rDNA cluster on medium-sized bivalent **h, i** *U. satunini* (2n = 31). Bar = 10 µm.

Table 1 shows the chromosome number (2n) and morphology of chromosomes (Fundamental Number = FN, the number of chromosome arms including of the X chromosome) of the Pholidopterini species by combining new data with previously published information (Warchałowska-Śliwa 1988, Warchałowska-Śliwa et al. 2005). All analyzed species show the same sex determination system X0 (male) and XX (female). All species of the genera *Eupholidoptera*, (Fig. 1a–c), most *Parapholidoptera* (Fig. 1d–l) (except P. aff. syriaca), six out of the 16 taxa of *Pholidoptera* (Fig. 2a-c) and one *Uvarovistia* species have 2n = 31 in male and 32 in female (Fig. 2h, i). Fifteen pairs of acrocentric autosomes can be subdivided into the three size groups: one long, five medium and nine short. On the other hand, in Parapholidoptera aff. syriaca (Fig. 1j–l) and in 10 out of 16 taxa of *Pholidoptera* (Fig. 2d–g), the complement is reduced to 2n = 29 (male) and 30 (female) as a result of one Robertsonian fusion (Warchałowska-Śliwa 1998). In this case, the bivalents may be classified according to size as one long submetacentric pair, four medium and nine short acrocentric pairs. Often, minor length differences in chromosome pairs cause problem with precise identification. In most Pholidopterini taxa, the X chromosome was acrocentric, except for the bi-armed X chromosomes in *Parapholidoptera grandis* (Karabag, 1952) (Fig. 1g–i) and *P. castaneoviridis* (Brunner von Wattenwyl, 1882) (not shown). B chromosome, a supernumerary element to the standard karyotype, was found in one *Parapholidoptera distincta* (Uvarov, 1921) male, being acrocentric and meiotically stable and similar in size to the short autosomes (Fig. 1f).

Cytogenetic maps of 18S rDNA were obtained for six *Eupholidoptera*, six *Parapholidoptera*, 15 *Pholidoptera* taxa and one *Uvarovistia* species. In each of the species, FISH revealed a single cluster of rDNA (per haploid genome), located on one autosome pair (Table 1). Its location was observed at mitotic metaphases or one bivalent from diakinesis to metaphase I. In all taxa with 31 (male) and 32 (female) chromosomes, 18S rDNA loci with different intensity signals were located in the interstitial area of the chromosomes – closer to their paracentromeric region (Figs 1a, b; 2a,c) or in the middle of probably the 3rd or 4th bivalent (Fig. 1g, j) or of 4/5th bivalent in *Uvarovistia satunini* (Uvarov, 1934) (Fig. 2h). On the other hand, in most taxa with 29/30 chromosomes, a high-intensity signal located in the paracentromeric region was found on the bi-armed first pair of autosomes (Fig. 2d, e); only in *Pholidoptera brevipes* Ramme, 1939 it appeared in the interstitial region in 3rd/4th bivalent (Fig. 2g).

Two species of *Parapholidoptera* and one *Pholidoptera* exhibited heteromorphism in the rDNA-FISH signal and NOR in terms of the size and presence/absence between homologous chromosomes (indicated with an asterisk in Table 1). In both *Parapholidoptera grandis* (Fig. 1g) and *Pholidoptera pustulipes* (Motschulsky, 1846) (Fig. 2b, c), one of the males analyzed showed size variation for the 18S rDNA clusters, whereas presence/absence heteromorphism was observed in Parapholidoptera prope syriaca (Fig. 1j, k). The signals produced by (TTAGG)*_n_* FISH occurred at the distal ends of each chromosome in most the taxa analyzed (invisible in some species, probably due to a technical error or to a low number of copies of telomeric repeats) (Figs 1g, j; 2h). These signals were not observed in the centromere region of bi-armed chromosomes.

Generally, regardless of the number of chromosomes in karyotype, after both C-band staining and fluorochrome double-staining, chromosome regions showed a similar pattern in analyzed species in terms of constitutive heterochromatin. Most species had heterochromatin blocks in the paracentromeric region with thin C-bands, very weakly staining with DAPI and CMA_3_ (CMA_3_-) negative (not shown). Only in the first chromosome pair of *Parapholidoptera grandis*, thick C-bands in paracentromeric and distal regions were visualized with both DAPI-positive (AT-rich) and CMA_3_-positive (GC-rich) signals (not shown). In this case, a heterochromatin heteromorphism in respect to the pattern of C-bands (Fig. 1h) and fluorochrome bands (not shown) was observed between individuals. However, in all analyzed Pholidopterini, thin or thick C-band, DAPI- and CMA_3_+ band were located interstitially on the 3rd/4th (Fig. 1c) or 4/5th bivalent in species with 2n = 31, 32 and in *Pholidoptera brevipes* with 2n = 29, as well as in the paracentromeric region on the long bi-armed autosome pair in species with 2n =29, 30 (not shown).

## Discussion

Up to now, cytotaxonomic studies of the Palaearctic Tettigoniinae in more than 60 species out of 22 genera, including 30 species of 11 genera from Europe showed that the most of species had 31 acrocentric chromosomes. However, in *Pholidoptera macedonica* and *Ph. aptera*, the chromosome number is reduced to 29 with one submetacentric long pair (Warchałowska-Śliwa 1988, 1998, Warchałowska-Śliwa et al. 2005).

Representatives of four Pholidopterini genera examined in this study have two different male karyotypes including 31 or 29 chromosomes, respectively, and the same sex determination system. The diploid number 2n = 31 (male) of all species belonging to *Eupholidoptera* and *Uvarovistia* (only one species was analyzed) as well as to *Parapholidoptera* (excluding Parapholidoptera prope syriaca) corroborates previous studies, which revealed that most species of the Palaearctic Tettigoniinae were characterized by such basic/ancestral karyotype (e.g. for review see Warchałowska-Śliwa 1998). Additionally, a pericentric inversion modifying the position of the centromere has changed the morphology of the ancestral acrocentric X chromosome to the biarmed X in two *Parapholidoptera* and two *Pholidoptera* species. A similar type of translocation was reported in some other species of Tettigoniinae (Warchałowska-Śliwa 1998), Phaneropterinae (Warchałowska-Śliwa et al. 2011) and Bradyporinae (e.g. Warchałowska-Śliwa et al. 2013a). Further analysis is required to confirm this assumption of polymorphism of such chromosome changes.

The ancestral chromosome number is reduced to 2n = 29 in almost half of the analyzed *Pholidoptera* species and only one *Parapholidoptera* species (P. prope syriaca) as a result of one Robertsonian translocation (the first autosome pair in the set becomes submetacentric). This reduction appears to be more frequent in the chromosomal evolutionary history of the subfamilies Tettigoniinae and Bradyporinae (e.g. White 1973, Hewitt 1979, Warchałowska-Śliwa 1988, 1998, Grzywacz et al. 2017 in press).

B chromosomes are frequent in orthopterans, especially in superfamilies Pyrgomorphoidea, Grylloidea, Acridoidea, Tetrigoidea (e.g. Palestis et al. 2010) while rare and often unstable in Tettigonioidea, (e.g. Warchałowska-Śliwa et al. 2005, 2008, Hemp et al. 2010a, 2015b). The origin of B chromosome in *Parapholidoptera distincta* is currently not clearly known and needs a comparison of the DNA sequences shared by both autosomes and Bs.

In the Pholidopterini chromosomes described in this paper, one 18S rDNA FISH locus (per haploid genome) coincides with a single active NOR detected by Ag-NO_3_ staining and with a C-band/CG-rich segment, independently from the number of chromosomes in the set. However, in analyzed species/subspecies two different patterns of the location of rDNA/NOR were observed. A single bivalent carrying 18S rDNA clusters in the interstitial region on a medium-sized chromosome (3rd/4th pair) seems to be typical feature of the representatives of *Eupholidoptera*, some *Pholidoptera* and *Parapholidoptera* taxa with 31 chromosomes as well as Parapholidoptera prope syriaca with chromosome number reduced to 29 in male. The 18S rDNA location on the 4/5th pair of *Uvarovistia satunini* might represent a derived aspect. Additionally, some structural rearrangements, e.g. a small inversion, may have been involved in changes of the rDNA location in two *Parapholidoptera* species. By contrast, in seven species/subspecies with 29 chromosomes, one paracentromeric rDNA site, exhibited a unique rDNA distribution pattern in the long bi-armed autosome. The distribution of rDNA loci/active NORs show that a Robertsonian translocation between the first pair and medium sized pair of chromosome-bearing rDNA cluster (probably 3rd/4th) has reduced the chromosome number from 2n = 31 (FN=31, 32) to 29 (FN=31, 32).

The presence of interstitial rDNA loci on a single bivalent (acrocentric or bi-armed) has been observed in the same subfamily in the tribe Platycleidini (Grzywacz et al. 2017 in press) and in some Bradyporinae karyotypes where a reduction of the chromosome number as the result of tandem fusion or Robertsonian translocation was suggested (Warchałowska-Śliwa et al. 2013a). These observations show that frequently rDNA/NOR-bearing chromosomes take part in karyotype rearrangements. The rDNA of terminal NORs is considered to be involved in Robertsonian translocations and in this case either very small centric or acentric fragments may be eliminated during subsequent mitotic or meiotic divisions or be undetectable by microscopic visualization techniques (Lysák and Schubert 2013). The lack of interstitial (TTAGG)*_n_* sequences (tDNA-FISH signal) in the centromeric region of bi-armed chromosomes, observed in some Pholidopterini species, could probably be due to a loss of telomeric repeats during karyotype evolution.

The presence of paracentromeric 18S rDNA cluster on a single bivalent was previously observed in different size chromosomes of other tettigoniids: in European (e.g. Warchałowska-Śliwa et al. 2013b) and African (Hemp et al. 2010b, 2013, 2015a) Phaneropterinae likewise in European Saginae (Warchałowska-Śliwa et al. 2009) and Bradyporinae (Warchałowska-Śliwa et al. 2013a), as well as in some grasshoppers (e.g. Cabral-de-Mello et al. 2011b). Probably, 18S rDNA cluster in Pholidopterini (present results) and Platycleidini (Grzywacz et al. 2017 in press) is syntenic in the interstitial position on chromosome pair 3rd/4th in karyotype with 31 chromosomes. This localization suggests occurrence of chromosome reorganization that probably occurred in their common ancestor by inversion causing displacement of the 18S rDNA cluster from paracentromeric to an interstitial position.

Generally, the Pholidopterini are characterized by chromosomes with a small amount of constitutive heterochromatin in the paracentromeric region and interstitially located C-bands in a medium sized autosome. In most of species and genera belonging to European Tettigoniinae, thin paracentromeric C-bands were uniformly present in chromosomes, but the C-banding patterns and distribution of interstitial and telomeric heterochromatin are usually found to vary among genera and sometimes between species of one genus (e.g. Warchałowska-Śliwa et al. 2005). In most of analyzed species only the interstitial region of the 3rd/4th autosome pair showed thin C-bands and bright CMA_3_ (DAPI-negative) CG-rich segments (except the first chromosome pair of *Parapholidoptera grandis* with thick C-bands/DAPI+/CMA_3_+ signals). Our results demonstrate a coincidence between the location of rDNA loci and active NOR and C/GC-rich heterochromatin regions, which indicates the presence of multiple repetitive DNA sequences. In a few Pholidopterini species, the size of the rDNA hybridization signals on homologous pairs of autosomes (Table 1; indicated by an asterisk) and the pattern of heterochromatin distribution have revealed size heteromorphism in the NOR and C-bands. Present results indicate different intensities of hybridization signals on the autosomes (3rd/4th) of *Parapholidoptera grandis* and *Pholidoptera pustulipes*, reaching an extreme case in Parapholidoptera prope syriaca (only one chromosome showed a hybridization signal). These differences, detected by e.g. Ag-NOR banding technique, suggested the occurrence of a polymorphism in the number of rDNA sequence copies. Similar heteromorphism has been observed in other tettigoniids (e.g. Warchałowska-Śliwa et al. 2013a,b, Grzywacz et al. 2014a,b) as a result of different mechanisms, i.e. homologous translocation, unequal crossing-over, homologous translocation or specific rearrangements of repetitive DNA families (e.g. Bressa et al. 2008, Cabrel-de-Mello et al. 2011b).

In conclusion, the results described in this paper demonstrate the usefulness of molecular techniques as tools for better understanding of chromosomal organization and evolutionary history in the tribe Pholidopterini. This study show that the karyotypes of the species analyzed have undergone evolution including changes in chromosome number and morphology by one Robertsonian translocation and sporadically inversion in the X chromosome. The location of the single rDNA/NOR coinciding with C-band/CG-rich segment in the genus *Pholidoptera* suggests that reduced chromosome number from 31 to 29 in male resulted from a Robertsonian translocation between two pairs of autosomes, one carrying the rDNA/NOR. Thus, FISH for identifying the location of 18S rDNA has proved to be a good marker for distinguishing species in this genus. On the other hand, the tendency of interstitial distribution of repeated DNA sequences may represent a cytogenetic marker for distinguishing some genera in Tettigoniinae. In contrast, interspecific autosomal differentiation has involved minor differences concerning the heterochromatin composition and distribution obtained by C-banding and fluorochrome staining. Furthermore, the additionally detected taxon-specific karyotypic differences in Pholidopterini need to be compared with phylogenetic data to get a clearer idea of their importance both for understanding the karyotype evolution and speciation within this group.
